# A Multimodal Imaging-Supported Down Syndrome Mouse Model of RSV Infection

**DOI:** 10.3390/v15040993

**Published:** 2023-04-18

**Authors:** Birger Tielemans, Lander De Herdt, Emilie Pollenus, Emiel Vanhulle, Laura Seldeslachts, Fopke Marain, Flore Belmans, Kaveh Ahookhosh, Jeroen Vanoirbeek, Kurt Vermeire, Philippe E. Van den Steen, Greetje Vande Velde

**Affiliations:** 1 Biomedical MRI Unit/Mosaic, Department of Imaging and Pathology, KU Leuven, 3000 Leuven, Belgium; lander.deherdt@kuleuven.be (L.D.H.);; 2 Laboratory of Immunoparasitology, Department of Microbiology, Immunology and Transplantation, Rega Institute for Medical Research, KU Leuven, 3000 Leuven, Belgium; 3 Laboratory of Virology and Chemotherapy, Department of Microbiology, Immunology and Transplantation, Rega Institute for Medical Research, KU Leuven, 3000 Leuven, Belgium; 4 Laboratory of Respiratory Diseases and Thoracic Surgery (BREATHE), Department of Chronic Diseases and Metabolism, KU Leuven, 3000 Leuven, Belgium; 5 Radiomics (Oncoradiomics SA), 4000 Liege, Belgium; 6 Centre for Environment and Health, Department of Public Health and Primary Care, KU Leuven, 3000 Leuven, Belgium

**Keywords:** RSV development, Down syndrome, Ts65Dn mouse model, non-invasive imaging, respiratory infections, DS immunity

## Abstract

Individuals with Down syndrome (DS) are more prone to develop severe respiratory tract infections. Although a RSV infection has a high clinical impact and severe outcome in individuals with DS, no vaccine nor effective therapeutics are available. Any research into infection pathophysiology or prophylactic and therapeutic antiviral strategies in the specific context of DS would greatly benefit this patient population, but currently such relevant animal models are lacking. This study aimed to develop and characterize the first mouse model of RSV infection in a DS-specific context. Ts65Dn mice and wild type littermates were inoculated with a bioluminescence imaging-enabled recombinant human RSV to longitudinally track viral replication in host cells throughout infection progression. This resulted in an active infection in the upper airways and lungs with similar viral load in Ts65Dn mice and euploid mice. Flow cytometric analysis of leukocytes in lungs and spleen demonstrated immune alterations with lower CD8+ T cells and B-cells in Ts65Dn mice. Overall, our study presents a novel DS-specific mouse model of hRSV infection and shows that potential in using the Ts65Dn preclinical model to study immune-specific responses of RSV in the context of DS and supports the need for models representing the pathological development.

## 1. Introduction

Worldwide, acute lower respiratory tract infections (LRTIs) are a major cause of hospital admissions and mortality in children below the age of 5 years [1]. Respiratory syncytial virus (RSV) is the most common single cause of respiratory hospitalization and LRTIs in infants [2,3], affecting nearly 100% of children <2 years of age [4]. Acute RSV infections can result in a spectrum of complications ranging from mild upper airway infections to severe bronchiolitis and pneumonia in infants, elderly and immunodeficient patients, requiring hospitalization and mechanical ventilation in up to 3% of cases [5,6]. Apart from immunodeficiency, other known risk factors for severe RSV disease are prematurity, congenital heart disease (CHD), and chronic lung disease [4,7,8]. Individuals with Down syndrome (DS) present several of these risk factors, including CHD and immunodeficiency, making DS an independent risk factor for severe RSV-associated LRTIs [9] and an important comorbidity for RSV-related deaths (odds ratio = 13.01) [10].

The elevated incidence of RSV infection observed in individuals with DS categorizes trisomy as an independent risk factor for severe lower RSV-associated LRTIs [9]. Indeed, during the Spanish RSV season of 2012, a cohort study including 97 children with DS and 70 children without DS (<1 year) showed the hospitalization of 10 children with DS compared to one child without DS due to RSV as a single pathogen, indicating a higher severity of RSV infections in DS [11]. Later studies confirmed these findings, reporting that RSV infections in children with DS resulted in a 6.5 times higher hospitalization rate, prolonged length of hospitalization, and clinically more severe illness with an increased need for mechanical ventilation, as compared to the euploid population [12,13]. The current hypothesis is that the high comorbidity of DS in severe RSV-induced LRTIs can be related to a plethora of DS-related structural airway anomalies, congenital cardiopulmonary dysfunctions, and altered immune response [12,14], but the exact mechanisms are unknown.

Structurally, stereotypical craniofacial malformations and upper airway anomalies, including macroglossia, pharyngeal hypotonia, adenoid hypertrophy, tracheal stenosis, and bronchomalacia [15,16], increase the risk of airway obstruction which may in turn result in a lower viral clearance and pathogen accumulation [17,18,19]. Functional alterations, including cilia perturbations and a deficient respiratory mucosal clearance, may result in pathogen accumulation in the upper airways [20,21,22]. Moreover, individuals with DS are known to have delayed lung development and suffer from pulmonary hypoplasia [20,21]. Therefore, postnatal DS lungs are characterized by an immature lung, indicated by lower organ size, decreased airway branching pattern and number of alveoli [20], resulting in a lower lung function and lung capacity to compensate for infection-related hypoxia [23]. Simultaneously, the pulmonary vascular development shows signs of immature development with a double capillary network and bronchopulmonary anastomoses in DS [21], causing an increased risk of developing significant cardiovascular pathologies, including CHD or pulmonary arterial hypertension (PAH), thereby pressuring the respiratory system [24,25,26]. In addition, people with DS suffer from an impaired immune response characterized by impaired haematopoiesis, resulting in low proliferation and differentiation rates of immune cells together with interferon hypersensitivity [20,27]. These alterations lead to reduced responsiveness of neutrophils, monocytes and natural killer (NK) cells, whereas B and T cells present signs of senescence [28,29,30,31], increasing the likelihood of auto-immune disorders, lower viral clearing, and a clinically worse outcome during infections [14,27].

Despite their clinical relevance and importance, the exact impact of each of these structural, functional and immunological alterations on the increased morbidity and mortality in the DS population upon RSV infections is unknown, as too few preclinical studies have focused on this specific high-risk group [32,33,34]. Due to its clinical significance, there is an urgent need to understand the DS-specific pathophysiological mechanisms behind a RSV-induced LRTI to further optimize treatment regimen and therapeutic outcome in this specific patient cohort.

Indeed, there is no effective antiviral therapy or human RSV (hRSV) vaccine approved yet, and several vaccines, long-lasting monoclonal antibodies, and antiviral therapies that are in advanced development, and ongoing clinical trials, lack the inclusion of individuals with DS [35,36,37]. It is nevertheless relevant to study prophylactic and therapeutic antiviral strategies in this specific population as the altered immune system in individuals with DS may result in a hampered vaccine or treatment response and may require a DS-specific vaccine or treatment strategy. Thereby, we need to consider the main challenges when developing vaccines for paediatric use, which include: (I) the need to vaccinate at an early age when the humoral immune response of the infant has not been fully developed and mainly maternal antibodies are present; (II) the difficulty of reliably demonstrating their safety and efficacy in preclinical studies; and (III) the lack of animal models that fully reproduce the pathogenesis of hRSV infection in humans. Most frequently used animal models of hRSV include non-human primates, lambs, cotton rats and mice [38]. However, while there are well-described models for DS that recapitulate most of the cognitive and craniofacial phenotypes in DS, there is no DS-specific RSV LRTI model to investigate the DS-specific pathophysiological mechanisms. Therefore, the effectivity of drugs and medicines in this high-risk group remains unknown [39,40]. To investigate the effects of the structural, functional, and immunological alterations on the increased incidence of RSV-induced LRTI in a DS-specific context, a properly characterized preclinical model is required.

To alleviate this need, this study aims to develop a DS-specific murine model of RSV infection which allows non-invasive and longitudinal follow-up of genotype-dependent disease progression. Using the most commonly used Ts65Dn mouse model, we were able to longitudinally track viral replication and presence of infection in wild-type and Ts65Dn mice using non-invasive bioluminescence imaging (BLI). In addition, we measured viral load four and seven days after infection. We performed daily micro-computed tomography (micro-CT) to assess disease progression and detailed lung function measurements four and seven days after infection to investigate the effect of infection on a battery of lung function and airway sensitivity. Finally, we characterized the pulmonary and systemic immune response at baseline and upon RSV infection using flow cytometry. In this study, we established a fully characterized multimodal imaging-based Ts65Dn hRSV mouse model, enabling longitudinal structural, functional, and immunological studies of hRSV infections in DS.

## 2. Materials and Methods

### 2.1. Mouse Model

All animal experiments were approved by the Institutional Animal Ethics committee of KU Leuven (P149/2021). All animal experiments were carried out in compliance with national and European regulations. Six to seven-week-old male trisomic (Ts65Dn) mice (strain #: 005252) and euploid (wild type) littermates were obtained from the Jackson Laboratory (Bar Harbor, ME, USA) and kept in a conventional animal facility with controlled environmental conditions in individually ventilated cages and free access to food and water. 

### 2.2. Experimental Design

The mice were randomly assigned by genotype to experimental groups and were either sham infected with dPBS or infected with a luciferase-recombinant human respiratory syncytial virus (rhRSV-Luc) Long strain, engineered and kindly gifted by Rameix-Welti and colleagues [41]. The virus was propagated in Hep-2 cells according to a standard procedure to obtain high-titre viral stocks as described elsewhere [41].

**Figure 1:**
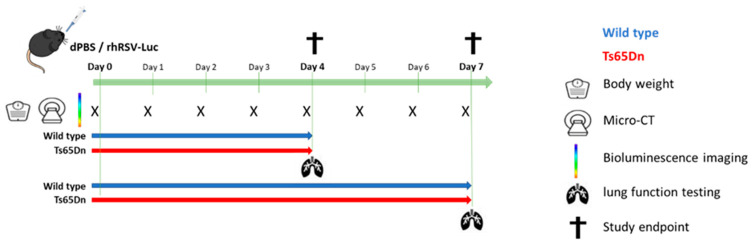
Schematic overview of the experimental setup. Six- to seven-week old wild type (blue) and Ts65Dn (red) mice received intranasal instillation with dPBS or rhRSV-Luc at d0. Mice were longitudinally monitored daily for body weight, with micro-computed tomography (micro-CT) and bioluminescence imaging (BLI). At study endpoint at four and seven days after infection, invasive lung function measures were performed, and tissue samples collected for immunological analyses. The number of animals ranged between four and six between the different groups (wild type vs Ts65Dn, sham infected vs rhRSV-Luc infected, 4 days p.i. vs 7 days p.i.).

Mice were anesthetized using 2 mL/min isoflurane and infected intranasally with 50 μL of dPBS containing vehicle (sham) or 1.7 × 10^6^ PFU/mL rhRSV-Luc. Mice were monitored daily for overall health, weighed for obtaining body weight, and imaged with bioluminescence imaging (BLI) and micro-computed tomography (micro-CT). The groups were sacrificed at the predefined endpoints, four or seven days after inoculation (Figure 1).

### 2.3. Micro-Computed Tomography (Micro-CT)

All animals were scanned daily using micro-CT as previously described [42], on a SkyScan 1278 (Bruker μCT, Kontich, Belgium) with the following scan parameters: 50 kVp X-ray source, 1 mm aluminum X-ray filter, 350 μA current, 150 ms exposure time per projection, 0.9° increments over a total angle of 220°, resulting in scan time of 3 min and radiation dose exposure of 60–80 mGy [43]. Reconstruction, visualization, analysis, and rendering of micro-CT data was performed with software from the manufacturer (NRecon, Data-Viewer, CTan, and CTvox). With an in-house trained convolutional neural network, the volume of interest (VOI) covering the entire lung was automatically delineated. Imaging-derived biomarkers of lung pathology (total lung volume (TLV), aerated lung volume (ALV), non-aerated lung volume (NALV), and their respective densities) were extracted by applying fixed threshold of −150 HU to separate ALV from NALV [44]. Calculations were performed using MATLAB (The MathWorks, Inc.; Natick, MA, USA).

### 2.4. Bioluminescence Imaging (BLI)

For in vivo BLI of RSV infected tissue, the thorax of the mice were depilated using depilatory cream at the start of the experiment and all animals were scanned daily thereafter. Ten minutes after intraperitoneal injection of 500 mg/kg sodium D-luciferin (50 mg/mL, Gold Biotechnology^®^, St Louis, MI, USA), mice were placed in a supine position in the IVIS Spectrum system (Perkin-Elmer, Hopkinton, MA, USA). A minimum of five consecutive images were acquired until maximal signal was detected with the following settings: exposure time 1 min, medium binning, F/stop of 1 and subject height of 1.5 cm [42,45]. Quantification of BLI data was performed with Living Image Software version 4.7.3 using a region of interest (ROI) of 6.9 cm^2^ covering the lungs and 3.0 cm^2^ covering the nasal area of each mouse. For each ROI, the maximum signal of total photon flux was used for further analysis.

### 2.5. Lung Function and Airway Hyperreactivity Measurements

Lung function parameters and airway hyperreactivity were assessed using the FlexiVent™ system (EMKA Technologies—SCIREQ, Montreal, QC, Canada), as previously described [43,46]. Briefly, mice were lethally anaesthetized through intraperitoneal injection of pentobarbital (120 mg/kg body weight, Dolethal^®^, Vetoquinol, Alcázar de San Juan, Spain). Tracheotomy was performed when the animal was sufficiently anaesthetized and showed no body reflexes. Next, an 18-gauge metal cannula was inserted. Spontaneous breathing was mimicked by quasi-sinusoidally ventilation with a tidal volume of 10 mL/kg and a frequency of 150 breaths/min. First, two deep inflations were performed to inflate the lungs to a pressure of 30 cmH_2_O to open the lungs. Next, airway responsiveness was evaluated by nebulizing increasing concentration of methacholine aerosols (0, 2.5, 5, 10, 20, and 40 mg/mL in saline) for five seconds. After the aerosol exposure, forced oscillation perturbation (Quick Prime-3, QP3), which generates forced oscillations with a frequency between 1 and 20.5 Hz during 3 s, was performed five times. Central airway resistance (Rn) is an average of the five measurements. After the QP3 sequence, a negative pressure forced expiration (NPFE) perturbation was executed to measure forced expiratory volume in 0.1 s (FEV_0.1_), in 0.2 s (FEV_0.2_) and forced vital capacity (FVC) at each methacholine concentration. When the coefficient of determination (COD) was lower than 0.9, the measurement was excluded. Tiffeneau-index0.1 was calculated using FEV_0.1_ and FVC (FEV_0.1_/FVC).

### 2.6. Bronchoalveolar Lavage

After lung function measurements, bronchoalveolar lavage (BAL) fluid was collected by flushing the lungs three times with 0.7 mL 0.9% NaCl solution (Braun, Diegem, Belgium).

### 2.7. Differential Cell Counts

BAL fluid was centrifuged for 10 min at 1000× *g* at 4 °C. The supernatant was collected and stored at −80 °C until further use, and the pellet was resuspended in 1 mL 0.9% NaCl solution. Total viable cell count was determined using a Trypan blue staining (BioWhittaker^®^ Lonza, Basel, Switzerland) and counted on a Bürker haemocytometer [47]. Cells were prepared for differential cell count by spinning 250 μL of cells (Cytospin 3, Shandon, TechGen, Zellik, Belgium) at 1400× *g* for six minutes on microscope slides. Slides were air-dried and stained (Diff-Quik^®^ method, Medical Diagnostics, Düdingen, Germany). For each sample, 250 cells were counted to determine the percentage of macrophages, lymphocytes, neutrophils, and eosinophils in BAL fluid.

### 2.8. Viral Load Quantification

After BALF collection, the inferior lung lobe was collected, snap frozen, and stored at −80 °C. Lungs were crushed and total RNA was extracted from lung homogenates using the RNeasy mini kit (Qiagen, cat n°74104) according to the manufacturer’s instructions. Viral load of hRSV was determined by reverse transcription quantitative real-time PCR (RT-qPCR; QuantStudio™5 Real-Time PCR System (Applied Biosystems, Foster City, CA, USA) of the N gene of rhRSV-Luc. Each reaction (20 µL) that contained 5 µL of sample (extracted vRNA), 1× TaqMan Fast Virus 1-step MasterMix 4× (Applied Biosystems, cat n°4444434), 0.2 µM of both forward (5′-TGCACACTAGCATGTCCTAAC-3′) and reverse (5′-GGCAGTAGAGTTGAAGGGATTT-3′) primers, and 0.1 µM probe (5′-TGCCCTGCACCATAGGCATTCATA-3′). All primers and probes were obtained from Integrated DNA Technologies (IDT, Iowa, USA). The following PCR run conditions were used: one cycle of 15 min at 50 °C and 2 min at 95 °C, followed by 40 cycles of 15 s at 95 °C and 45 s at 60 °C. Data analysis was performed using the QuantStudio™ Design & Analysis Software (version v1.5.2, Applied Biosystems). A standard curve was produced using 10-fold serial dilutions of plasmid DNA with known concentrations. All samples were run in duplicate.

### 2.9. Spleen Cell Isolation

Spleens were removed and collected in PBS + 2% fetal calf serum (FCS, Gibco) at 4 °C. Next, the spleen was mashed through a 70 µm nylon cell strainer (VWR, Leuven, Belgium) to obtain single cells. Red blood cells were lysed during treatment with 0.83% ammonium chloride (Acros organics, Geel, Belgium)/10 mM Tris (Sigma-Aldrich, Saint-Louis, MI, USA) solution with pH 7.2 at 37 °C. After washing with PBS + 2% FCS, cells were resuspended in PBS + 2% FCS and live cells were counted after 1/2 dilution in trypan blue (VWR) in a Bürker chamber.

### 2.10. Lungs Cell Isolation

The left lung and post-caval lobe were removed and collected in RPMI buffer (RPMI glutamax + 5% FCS + 1% penicillin/streptomycin) with 0.1% beta-mercaptoethanol at room temperature (RT). Using scissors, lungs were first minced into small chunks then incubated in digestion medium (2 mg/mL collagenase D and 0.1 mg/mL DNase I in RPMI buffer) for 30 min at 37 °C. Afterward, tissue chunks were further dissociated by passing through a needle and syringe, and fresh digestion medium was added for a second incubation at 37 °C for 15 min. Lung tissue was again dissociated with the syringe and centrifuged (5 min, 708× *g*, RT). The cell pellet was resuspended using 10 mM EDTA and further diluted in PBS + 2% FCS. After another centrifugation, cells were treated with 0.83% NH4Cl/ 10 mM Tris at 37 °C to lyse RBCs and passed through a 70 µm nylon cell strainer. After washing with PBS + 2% FCS, cells were resuspended in PBS + 2% FCS and live cells were counted after 1/2 dilution in trypan blue in a Bürker chamber.

### 2.11. Flow Cytometric Immune Cell Analysis

A total of 1.5–3 million cells per sample were plated into 96 well plates and washed with PBS. Cells were incubated with a viability dye, Zombie Aqua (1/1000; Biolegend, San Diego, CA, USA) or Zombie UV (1/1000; Biolegend), together with Mice Fc block (MACS Miltenyi Biotec, Bergisch Gladbach, Germany) for 15 min at RT in the dark. After washing twice with cold PBS + 2% FCS + 2 mM EDTA, the cells were stained, as previously described [48]. This staining included a panel of monoclonal antibodies (Supplementary Table S1) against surface markers dissolved in PBS with Brilliant stain buffer (BD Biosciences; Erembodegem, Belgium) for 20 min at 4 °C in the dark. For the myeloid cell panel, cells positive for lineage-specific markers (with CD3, CD19, and NK1.1 as markers) were excluded using a dump gate. Cells were transferred to FACS tubes, washed twice with PBS and fixated in PBS + 0.4% formaldehyde.

A sum of 100,000 (Lymphoid panel) or 200,000 live single cells (Myeloid panel) were analysed per sample with a BD Fortessa Flow cytometer (BD Biosciences). Gating strategy for myeloid and lymphoid panels for both lung and spleen are shown in Supplementary Figures S1 and S2. Data was analysed with FlowJo v10 software (FlowJo LLC, Ashland, OR, USA). The frequency of a specific cell type among live cells was multiplied by the total number of live cells counted using the Bürker chamber to calculate the absolute number of that cell type.

### 2.12. Statistics

All categorical data (RT-qPCR, baseline lung functions, and flow cytometry) were analysed using the non-parametric Kruskal-Wallis test, followed by a Mann-Whitney post-hoc test with Holm-Bonferroni correction (GraphPad Prism, version 8.0.2). Correction was applied within the same genotype or within the same infectious condition (sham infected, four days p.i., seven days p.i.), depending on the research question. This was achieved by making use of the SciPy (version 1.9.0) and scikit-posthocs (version 0.7.0) Python (version 3.9.12) packages.

Longitudinal data (weight loss, BLI, µCT, and lung function during methacholine challenge) were analysed by mixed-effect model analysis, followed by multiple comparison with Holm-Bonferroni correction, by making use of the lme4 (version 1.1.31), lmerTest (version 3.1.3), emmeans (version 1.8.2) R (version 4.2.1) libraries, and the rpy2 (version 3.5.1) Python package. Categorical plots show individual values and median bars. Longitudinal plots show the mean and the accompanying standard deviation. All plots were generated with Matplotlib (version 3.5.2), seaborn (0.12.1), and statannotations (version 0.4.4) Python packages or GraphPad Prism (version 8.0.2). Differences were considered significant if p was smaller or equal to 0.05, significance levels were ranked as follows: * *p* < 0.05, ** *p* < 0.01, *** *p* < 0.001, **** *p* < 0.0001.

## 3. Results

### 3.1. Ts65Dn Mice Develop Upper Airway and Lung rhRSV-Luc Infection

**Figure 2:**
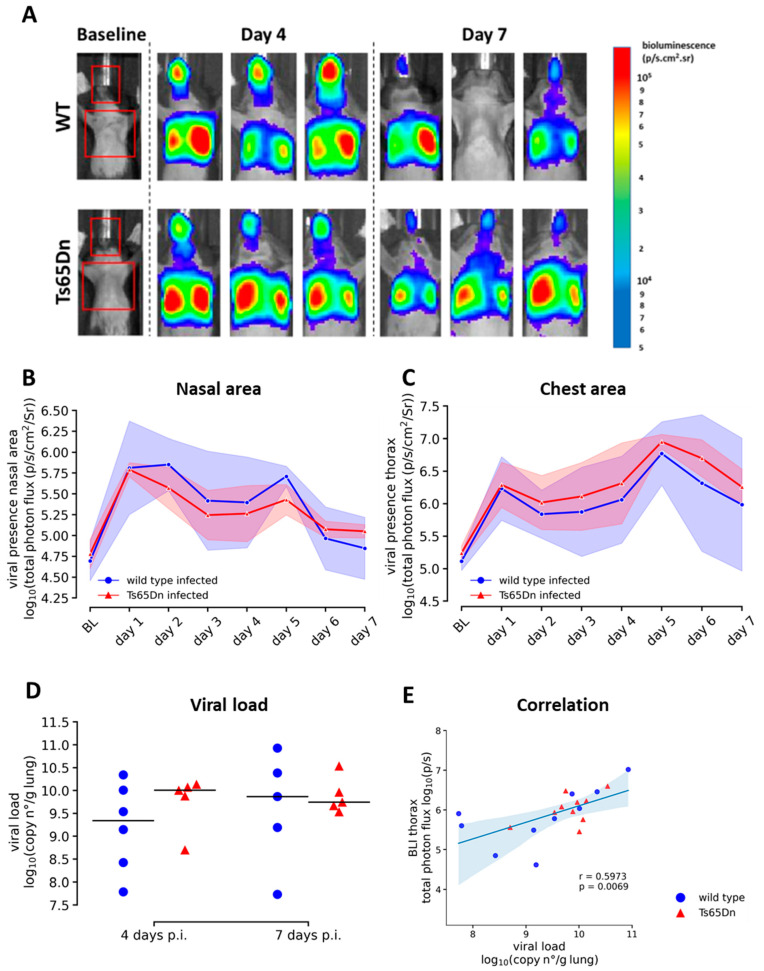
Airway and lung infection upon rhRSV-Luc in wild type and Ts65Dn mice. (**A**). Representative images of the ROI covering the lungs nasal area. Representative BLI images of mice at baseline, four days and seven days post rhRSV-Luc infection. (**B**) Total photon flux (log scale) from in vivo BLI based on a ROI covering the nasal area. (**C**) A ROI covering the thorax area of wild type (pooled 4 days infection N = 6 and 7 days infection N = 5) and Ts65Dn (pooled 4 days infection N = 5 and 7 days infection N = 5) mice. Data are shown as mean ± SD at each day. (**D**) Assessment of viral burden using RT-qPCR: graph representing the log10 viral copy number per gram lung tissue in wild type (blue circle) and Ts65Dn (red triangle) mice. Data are expressed as individual values and mean ± SD. (**E**) Correlation between viral load and BLI of the thorax area.

Wild type and Ts65Dn mice received intranasal instillation with a luciferase-encoding variant of hRSV (rhRSV-Luc) or dPBS control (Figure 1). To investigate the presence and establishment of the rhRSV-Luc infection, we performed daily BLI and measured viral load at endpoint four and seven days p.i. (Figure 1). Longitudinal BLI enabled spatiotemporal in vivo follow-up and development of rhRSV-Luc infection at a whole-body level (Figure 2A). Infections were characterized by a drastic increase (*p* < 0.0001) in total photon flux in the infected groups compared to sham infected and baseline measure indicating the presence of viral replication in infected host cells in the nasal area and thorax area of rhRSV-Luc infected mice (Figure 2A). Intranasal instillation resulted in an initial peak of viral replication one-day post-infection (p.i.) in the nasal area (Figure 2B), while infection peaked in the lungs five days p.i. (Figure 2C). The rhRSV-Luc infection spatially progressed similarly in Ts65Dn and wild type mice, without any significant differences in viral replication (Figure 2B,C). Correlation of endpoint measurements of viral load in infected lung tissue and viral replication in the thorax area confirmed our longitudinal observations on BLI (Figure 2E).

Remarkably, the interindividual variances on nasal and thorax BLI as well as on viral load are much lower in the Ts65Dn group compared to the infected wild type group at seven days p.i. (Figure 2B–D). In all Ts65Dn mice, the viral load was still high whereas with WT mice, we encountered an absence of viral replication on BLI at seven days p.i. in two wild type mice. A correlation analysis between viral load and thorax BLI signal demonstrated a better clustering and lower variation in the Ts65Dn mice compared to the wild type group (Figure 2E). Overall, our longitudinal analysis shows viral presence and replication in the lung, thereby indicating the presence of an active infection. This section may be divided by subheadings. It should provide a concise and precise description of the experimental results, their interpretation, as well as the experimental conclusions that can be drawn.

**Figure 3:**
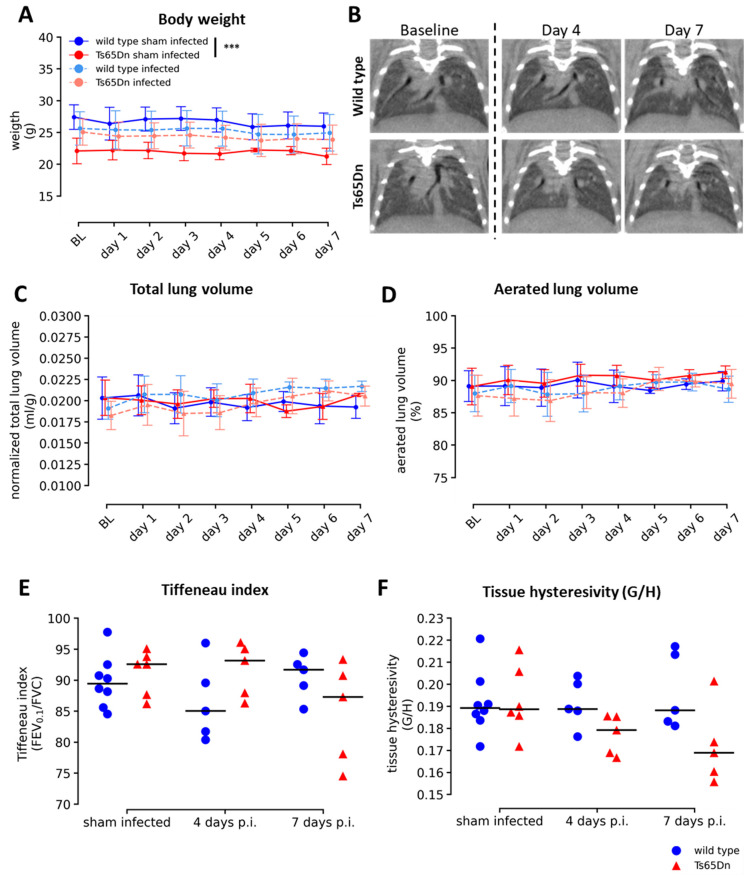
Overall pathological signs of a rhRSV-Luc infection in wild type and Ts65Dn mice. (**A**) Body weight was monitored in rhRSV-Luc and sham infected mice over seven days and expressed as mean with standard deviations. (**B**) Representative images of the frontal view of longitudinal lung micro-CT images from a wild type and Ts65Dn mouse at baseline, day four and day seven. (**C**) Representative graph showing the quantification of micro-CT-derived biomarkers showing total lung volume normalized to body weight. (**D**) Aerated lung volume expressed as percentage of TLV in wild type and Ts65Dn mice after intranasal instillation with dPBS or rhRSV-Luc. The number of mice were: wild type sham-infected (N = 7, 4 days p.i. N = 3, 7 days p.i. N = 4), Ts65Dn sham infected (N = 8, 4 days p.i. N = 4, 7 days p.i. N = 4), wild type infected (N = 11, 4 days p.i. N = 6, 7 days p.i. N = 5), and Ts65Dn infected (N = 10, 4 days p.i. N = 5, 7 days p.i. N = 5). (**E**) Pulmonary lung function data reflecting Tiffeneau index. (**F**) Tissue hysteresivity in sham-infected, four-day and seven-day infection in wild type (blue circle) and Ts65Dn (red triangle) mice, expressed as individual values and group median. *** *p* < 0.005.

The most common clinical scenario encountered in hRSV infection commonly presents as bronchiolitis and may progress to pneumonia in more severe cases. To investigate signs and severity of bronchiolitis and pneumonia in trisomic and euploid mice, we monitored daily health scoring and body weight, and performed non-invasive micro-CT. We observed that Ts65Dn mice had a lower initial body weight compared to WT mice. However, infection did not induce a loss in body weight in both genotypes (Figure 3A).

We did not observe signs of severe bronchiolitis or pneumonia, such as infiltrations, consolidations, pleural fluid, or bronchial dilation on the micro-CT data (Figure 3B). Extraction of micro-CT-derived biomarkers confirmed these results and revealed similar lung biomarkers at baseline and during the course of rhRSV-Luc infection in wild type and Ts65Dn mice up to seven days p.i. (Figure 3C,D).

To investigate the effect of a rhRSV-Luc infection on lung function, we extracted functional parameters by performing invasive lung function assessment at study endpoint. We found similar peak expiratory flow (PEF), forced vital capacity (FVC), forced expiratory volume in 0.1 s (FEV_0.1_) (data not shown), and Tiffeneau index for both wild type and Ts65Dn mice throughout infection (Figure 3E). Hysteresivity progressively decreased somewhat, although not significantly, in the Ts65Dn mice but not in wild type mice (Figure 3F). In general, these results are in line with the micro-CT-derived parameters which indicate that in both trisomic and euploid mice, the rhRSV-Luc infection does not progress into severe pneumonia.

As we observed active upper and lower rhRSV-Luc airway infection, albeit without severe bronchiolitis or pneumonia, we further investigated the airway inflammatory status to capture baseline genotype effects and changes upon rhRSV-Luc infection in the euploid and trisomic backgrounds.

**Figure 4:**
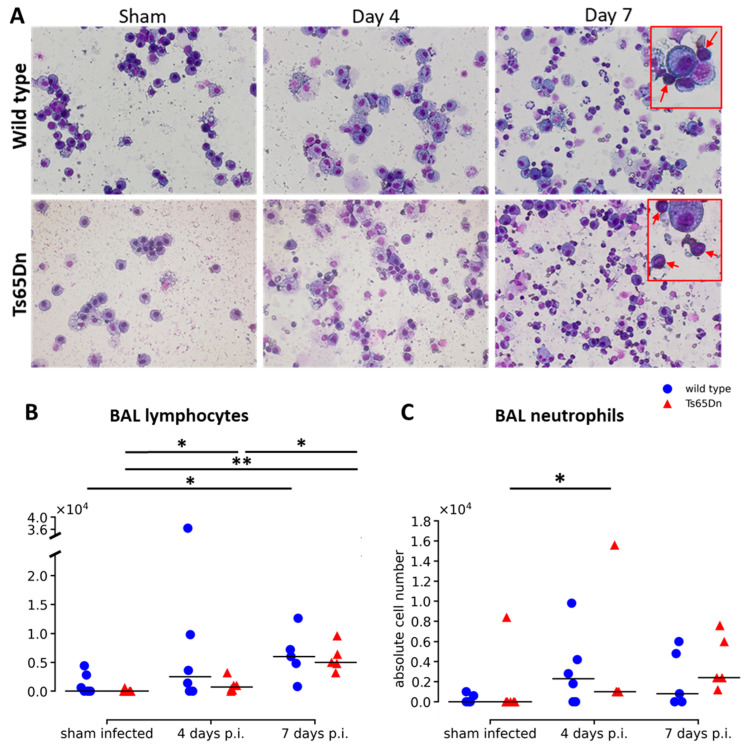
RSV infection causes airway inflammation. (**A**) Representative images of BAL from sham infected, four day infected, and seven day infected wild type and Ts65Dn mice with rhRSV-Luc indicating lymphocytes (red arrow). (**B**) Analysis of the number of lymphocytes. (**C**) Number of neutrophils in wild type (blue circle) and Ts65Dn (red triangle) mice at each time point and expressed as individual values and group median. * *p* < 0.05, ** *p* < 0.01.

BAL fluid was collected from sham and infected mice, four days p.i. and seven days p.i. and showed no differences in total cell counts and number of alveolar macrophages (Figure 4A and Supplementary Figure S3). At baseline, no differences were observed between WT and Ts65Dn mice in total number of cells, number of alveolar macrophages, lymphocytes, or neutrophils in the BAL fluid (Figure 4B,C and Supplementary Figure S3). Throughout infection, the number of lymphocytes in the BAL fluid increased over time of infection in comparison with the sham infected group, thereby indicating the presence of an immune response after rhRSV-Luc infection. In both genotypes, we observed a significant increase in total number of lymphocytes seven days p.i. (Figure 4B). The number of neutrophils peaked four days p.i. in the Ts65Dn mice (Figure 4C).

Taken together, detailed analysis of rhRSV-Luc infection showed airway inflammation in both Ts65Dn and wild type mice. We observed no differences at baseline between WT and Ts65Dn mice and an increase in lymphocytes throughout infection for both genotypes.

### 3.2. Increased Airway Inflammation by hRSV-Luc Infection Does Not Affect Airway Reactivity in Ts65Dn Mice

**Figure 5:**
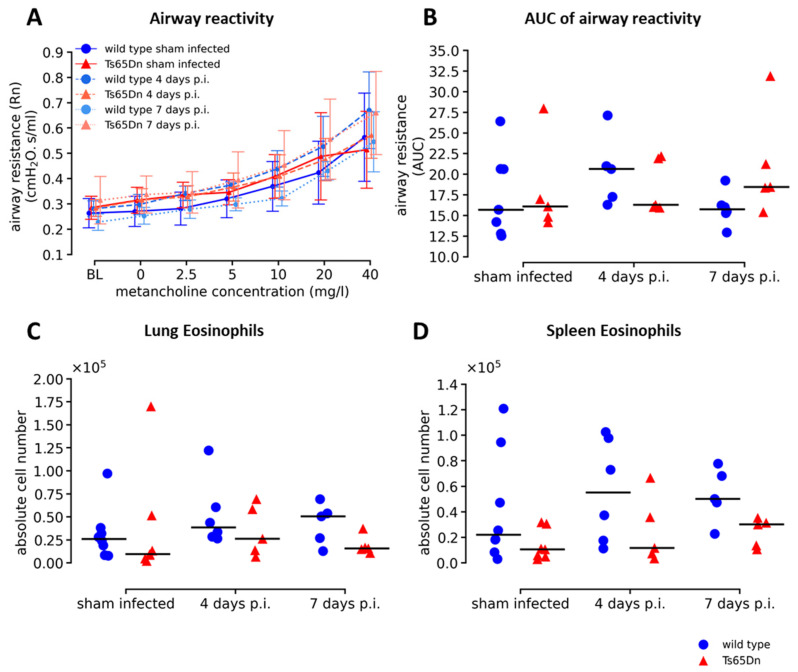
Airway responsiveness and asthmatic immune markers upon RSV infection. (**A**) Airway resistance was measured in response to increasing methacholine aerosol challenges (0–40 mg/mL) in wild type and Ts65Dn mice following sham, four days or seven days- rhRSV-Luc infection, and presented as group mean ± SD. The number of mice were: wild type sham infected (N = 8, 4days p.i. N = 4, 7days p.i. N = 4), Ts65Dn sham infected (N = 5, 4 days p.i. N = 2, 7 days p.i. N = 3), wild type infected (N = 10, 4 days p.i. N = 5, 7 days p.i. N = 5) and Ts65Dn infected (N = 9, 4 days p.i. N = 5, 7 days p.i. N = 4). (**B**) Calculations of the area under the curve (AUC) were presented for all groups and expressed as individual values with group median. The number of eosinophils in (**C**) lung and (**D**) spleen are measured using flow cytometry and presented as individual values, along with group median. Wild type (blue circle) and Ts65Dn (red triangle) with and without rhRSV-Luc infection were shown.

Since we observed active infection with an inflammatory phenotype in the airways with more lymphocytes in Ts65Dn and wild type mice over time of infection, we further investigated the functional impact of the rhRSV-Luc infection on the airways. We challenged sham infected, four and seven days p.i. mice with increased concentrations of methacholine, as airway irritant, to investigate alterations in airway reactivity. We observed no differences in hyperreactivity between wild type and Ts65Dn mice at baseline and throughout rhRSV-Luc infection, indicating no genotype differences (Figure 5A,B). Upon infection, both WT and Ts65Dn mice showed an equal increase in airway reactivity, thereby demonstrating no effect of a rhRSV-Luc infection on airway hyperreactivity (Figure 5A,B). Similarly, in parallel with the methacholine provocation test, we analysed the number of respiratory and systemic eosinophils as an asthmatic type 2 inflammatory biomarker and an important indicator for airway hyperreactivity following rhRSV infection (Figure 5C,D). We found equal numbers of eosinophils in lung and spleen throughout the course of infection that were not elevated compared to the sham infected groups. In addition, we observed no genotype differences between wild type and Ts65Dn mice in sham infected, four days, or seven days p.i. (Figure 5C,D).

These results indicate that although we observed an active infection of rhRSV-Luc inducing an immune response, this did not result in functional differences, as shown by a normal airway hyperreactivity and the absence of an asthmatic response upon rhRSV-Luc infection. Consequently, we observed no genotype differences regarding lung hyperreactivity and asthmatic response between wild type and Ts65Dn mice.

### 3.3. Lung Immune Status Shows Limited Genotype-Specific Responses upon rhRSV-Luc Infection

After initially investigating the impact of infection on airway function and airway immunity, we continued to investigate the response of the immune system upon a rhRSV-Luc infection in the lung parenchyma.

**Figure 6:**
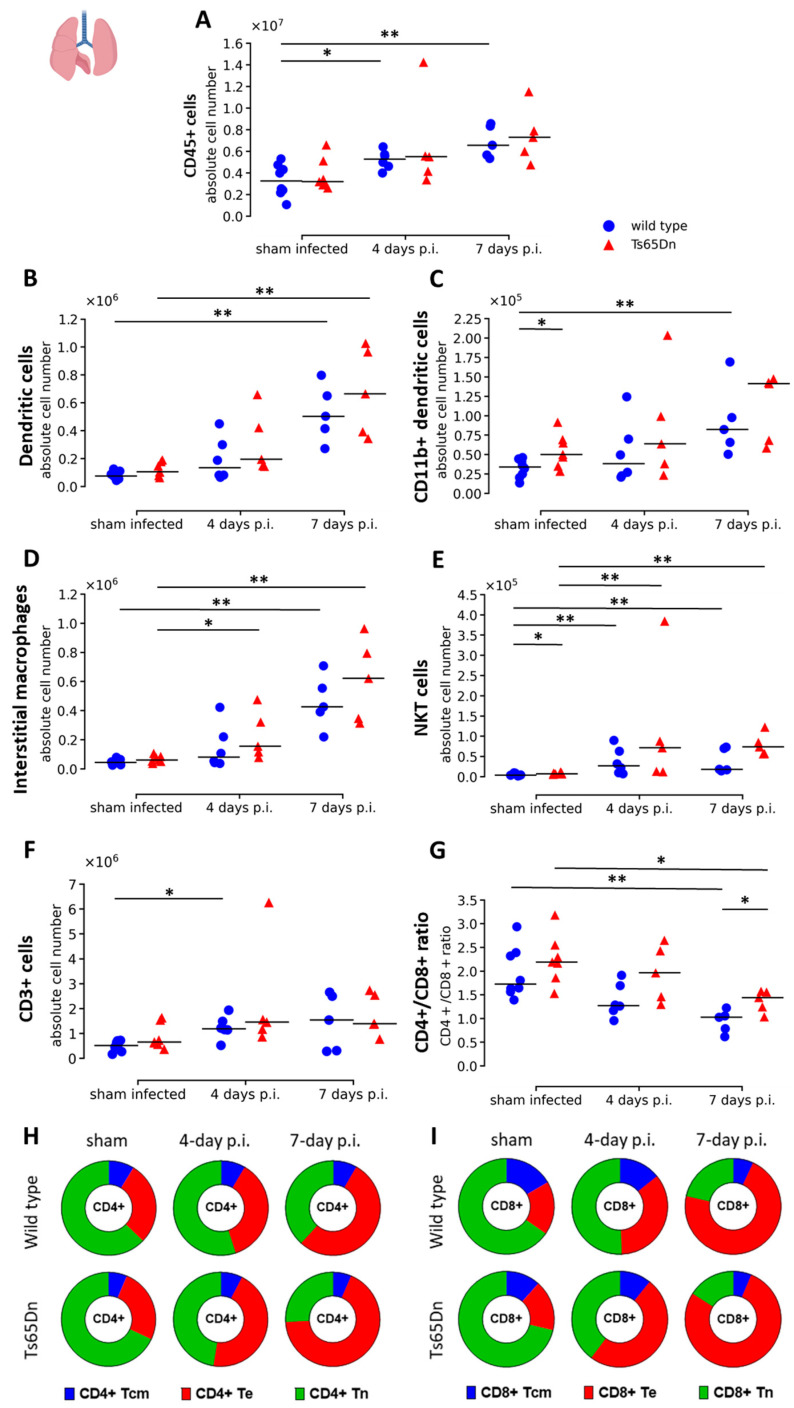
Flow cytometric analysis of pulmonary leukocytes in wild type and Ts65Dn mice after sham, four days or seven days rhRSV-Luc infection. (**A**) The absolute number of leukocytes (CD45+). (**B**) Lung dendritic cells (DCs; CD45+ Lin− SiglecF− MHC-II+ CD11c+). (**C**) CD11b+ DC (CD45+ Ly6G- SiglecF− MHC-II+ CD11c+ CD11b+). (**D**) Lung interstitial macrophages (CD45+ Lin− Ly6G- CD11bhi MHC-II+ CD64+ CD24−). (**E**) Lung NKT cells (CD45+ CD3+ NK1.1+) were presented as individual values and group median. (**F**) Lung T lymphocytes (CD3+) were presented as absolute number. (**G**) The ratio of CD4+ over CD8+ cells T cells and the proportions of, respectively, (**H**) CD4+ and (**I**) CD8+ Tcm (CD44+ CD62L+; blue), Te (CD44+ CD62L−; red), and Tn (CD44− CD62L+; green) of the total T-cell population are shown and presented as proportion of total CD4+ and CD8+ T cells. * *p* < 0.05, ** *p* < 0.01.

We observed no genotype differences in terms of CD45+ leukocytes at baseline. Infection resulted in a progressive leukocyte (CD45+ cells) response throughout infection in both genotypes. Higher levels of leukocytes were observed seven days p.i., suggesting increased recruitment even after peak of infection. Ts65Dn mice presented a similar trend as the wild type mice during infection with a similar trend in increased leukocytes seven days p.i. compared to baseline (*p* = 0.0530) (Figure 6A).

First, we analysed the differentiated immune cells involved in the innate immune response. Interestingly, CD11b+ dendritic cell and natural killer T cell (NKT cell) numbers were significantly higher at baseline in sham infected Ts65Dn mice compared to wild type mice (Figure 6C,E). This might indicate a more activated state of the immune system and potentially an increased immune response in Ts65Dn mice. Otherwise, no genotype alterations were observed at baseline, even in RSV-related cell types, such as eosinophils and neutrophils (Figure 5C and Supplementary Figure S4A). During rhRSV-Luc infection, we observed a progressive increase in number of dendritic cells, CD11b+ dendritic cells, interstitial macrophages, and NKT cells up to seven days p.i. compared to sham infected mice (Figure 6B–E). In combination with the results from BAL fluid, this suggests an effective innate immune response in the interstitial spaces of the lung. However, the initial genotype effect on CD11b+ dendritic cell and NKT cells observed in sham infected mice was lost during infection.

Next, we investigated the adaptive immune response in the lungs. Overall, we did not observe any genotype differences at baseline in the sham infected groups. During infection, higher numbers of CD3+ lymphocytes were observed in the lung tissue of the wild type group at four-days p.i. compared to sham infected mice (Figure 6F). The change in CD3+ lymphocytes is, in part, reflected by the increase in number of CD4+ and CD8+ T cells throughout infection. Analysis of the CD4+ and CD8+ T cells showed equal upregulation in both genotypes. However, the Ts65Dn mice tend to have an overall higher CD4+/CD8+ T-cell ratio compared to the WT mice, and this difference was significant after seven days of rhRSV-Luc infection (Figure 6G). When investigating the CD4+ and CD8+ subtypes, we observed a drastic increase in CD4+ and CD8+ T effector cells (Te) throughout infection (Figure 6H,I, Supplementary Figure S5). The genotype effect in CD4+/CD8+ T cell ratio observed at seven days p.i. is mainly due to a larger increase in number of CD4+ Te cells in the lungs of Ts65Dn mice compared to wild type mice, while the increase in CD8+ T effector cells was similar in Ts65Dn and wild type mice (Figure 6G–I, Supplementary Figure S5). Wild type and Ts65Dn mice showed similar number of B cells at baseline and throughout rhRSV-Luc infection (Supplementary Figure S4B).

In general, cellular analysis of lung tissue suggests that wild type and Ts65Dn mice clear the infection by a CD8+ T cell response and demonstrate higher levels of DCs, interstitial macrophages and NKT cells. Upregulation of CD4+ Te cells in the Ts65Dn mice may suggest a higher inflammatory response in the Ts65Dn mice.

### 3.4. A rhRSV-Luc Infection Results in Minor Splenic Innate Immune Activation

**Figure 7:**
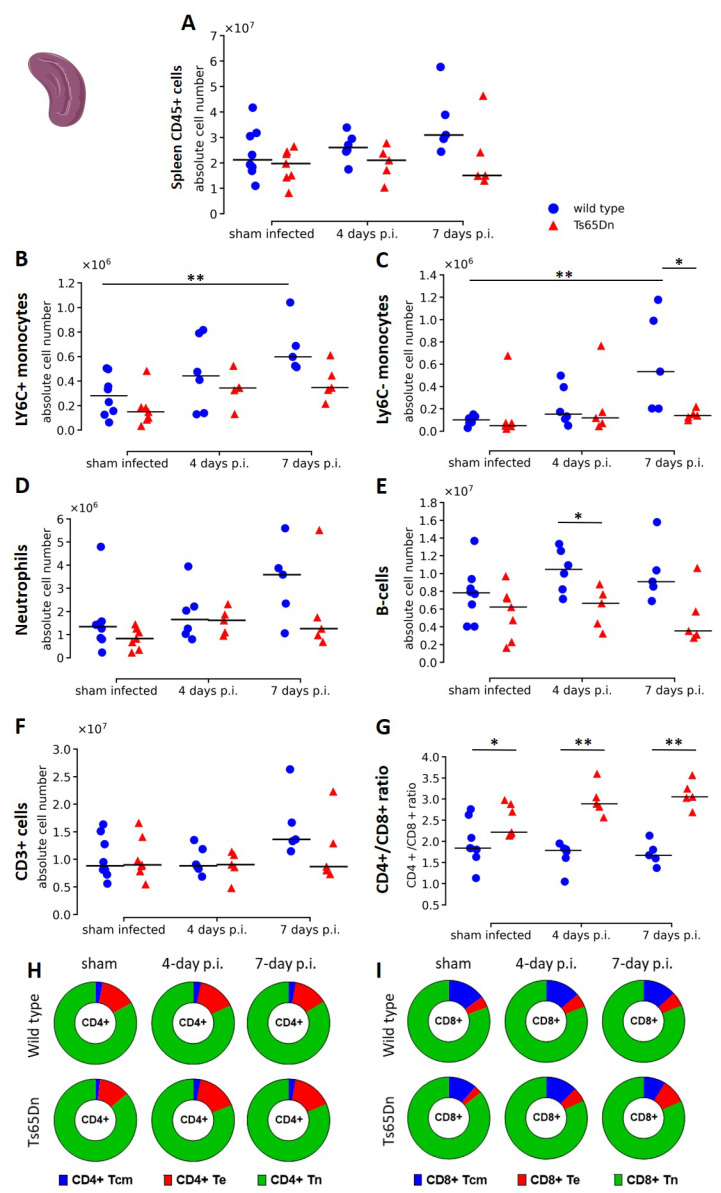
Immunological status of the spleen in wild type and Ts65Dn mice throughout rhRSV-Luc infection. (**A**) The absolute number of Leukocytes (CD45+). (**B**) Neutrophils (CD45+ Lin− CD11b+ Ly6G+), (**C**) B cells (CD45+ CD3− NK1.1− B220+). (**D**) Ly6C+ monocytes (CD45+ SiglecF- CD103- Ly6G- CD11bhi MHCII- Ly6C+). (**E**) Ly6C− monocytes (CD45+ SiglecF- CD103- Ly6G- CD11bhi MHCII- Ly6C-) were presented. (**F**) The absolute number of splenic CD3+ lymphocytes. (**G**) The ratio of CD4+ over CD8+ cells T cells were presented as individual values with group median. The proportions of, respectively. (**H**) CD4+. (**I**) CD8+ Tcm (CD44+ CD62L+; blue), Te (CD44+ CD62L−; red), and Tn (CD44− CD62L+; green) of the total T cell population are shown and presented as proportion of total CD4+ and CD8+ T cells. * *p* < 0.05, ** *p* < 0.01.

In parallel to the lung immune response, we investigated if rhRSV infection resulted in a systemic immune response by analysing the immune composition of the spleen at baseline and during rhRSV-Luc infection of wild type and Ts65Dn mice. At baseline, we observed similar levels of CD45+ leukocytes in the spleen of wild type and Ts65Dn mice (Figure 7A). During rhRSV-Luc infection, no effect on total number of CD45+ leukocytes was observed in comparison to baseline and between genotypes, indicating that there was no major splenic response upon intranasal rhRSV-Luc infection (Figure 7A).

Infection with rhRSV-Luc, resulted in a higher number of neutrophils, Ly6C- and Ly6C+ monocytes at seven days p.i. wild type mice compared to the sham infected mice. However, no increase was observed in the Ts65Dn mice (Figure 7B–D). During rhRSV-Luc infection, significantly higher Ly6C- monocytes at seven days p.i. and a trend of increased Ly6C+ monocytes (*p* = 0.0556) in wild type mice compared to Ts65Dn mice may suggest alterations in innate immune response in the Ts65Dn mice (Figure 7B).

When investigating the adaptive immune response, spleen CD3+ lymphocytes were similar in wild type and Ts65Dn mice in the sham infected groups (Figure 7F). However, we observed higher CD4+/CD8+ T cell ratios at baseline in the Ts65Dn mice compared to wild type mice, similar to the human situation (Figure 7G and Supplementary Figure S6). Further subclassification of CD8+ T cells showed equal numbers of naïve CD8+ T cells, effector CD8+ T cells and central memory CD8+ T cells (Figure 7I and Supplementary Figure S6).

During infection, the total number of CD3+ lymphocytes remained constant, indicating that the rhRSV-Luc infection induces only a local immune response in the lungs (Figure 6G and Figure 7G). However, the CD4+/CD8+ T cell ratio increased in the Ts65Dn mice along rhRSV-Luc infection but not in wild type mice (Figure 7G). This was attributed by higher CD8+ T cell numbers in spleen of wild type compared to Ts65Dn mice throughout rhRSV-Luc infection (Supplementary Figure S6). While the CD8+ Tn cells increased in the wild type mice, the absolute number of CD8+ Tn cells remained constant in the Ts65Dn mice (Figure 7I). Additionlly, the number of Tcm cells increased along infection in the wild type mice but not in the Ts65Dn mice and add to the total CD8+ genotype differences (Figure 7I and Supplementary Figure S6). In combination with lower B cell counts at four days infected Ts65Dn mice compared to wild type mice (Figure 7E), these results suggest that the Ts65Dn genotype is potentially more susceptible to recurrent infections.

## 4. Discussion

In this study we report the full characterization of a hRSV-induced respiratory infection in the Ts65Dn mouse model for DS. Our data show an effective host cell rhRSV-Luc infection with an active viral replication and indications of immunological alterations and increased level of local BAL fluid lymphocytes. We hereby deliver the first imaging-supported model of a rhRSV-Luc infection in the Ts65Dn DS mouse model, with full characterization of lung structure, function, and immunological phenotype throughout infection. 

### 4.1. In Vivo Imaging of a rhRSV-Luc DS Model Reveals Progressive Active Airway Infection without Severe Pneumonia

Using BLI, we demonstrated that a hRSV infection quickly develops in the upper respiratory airways (i.e., the nasal cavity that is first exposed to the virus), followed by increased viral replication in the lower respiratory system with a peak infection around five days, which is similar to the human situation [49]. We show a good correlation between viral replication assessed by in vivo BLI of the thorax and by RT-qPCR of viral RNA copy numbers in infected tissue, as previously described in BALB/c mice [41]. In addition, we encountered an absence of viral replication on BLI at seven days p.i. in two wild type mice. In contrast, all Ts65Dn mice show similar levels of viral replication with lower interindividual differences.

When analysing the phenotype throughout a rhRSV-Luc infection, we observed the presence of airway inflammation without any signs of pneumonia or bronchiolitis on micro-CT. In humans, a RSV infection is often considered non-harmful and self-limiting in most cases, only causing pneumonia in the most severe cases [49]. Mainly in children under 2 years, RSV infection may result in development of bronchiolitis or pneumonitis due to immature lung development and immunity. In this murine model, the differences in genotype did not result in a more severe clinical manifestation in the Ts65Dn mice.

It has been postulated that exposure to RSV infection early in life can lead to increased susceptibility to recurrent allergic wheezing and asthma during the following years. We investigated the effect of a respiratory RSV infection on lung function and development of an asthmatic phenotype [50]. However, there is no consensus on the prevalence of asthma in the DS population [51]. While some studies conclude that individuals with DS are unable to perform lung function testing due to cognitive disability, a clinical study by Da Silva et al. used healthy adult men with DS and showed a reduced pulmonary function compared to healthy controls and individuals with an equivalent IQ as individuals with DS [52]. In our Ts65Dn mouse model, we did not observe an altered lung function at baseline and throughout a rhRSV-Luc infection. A rhRSV-Luc infection did not alter airway hyperreactivity implicating the absence of any asthmatic phenotype or response to infection in our trisomic mouse model, confirmed by similar levels of eosinophils. Additionally, the age at initial infection has been reported to play a critical role, where younger mice experience increased airway hyperreactivity, enhanced mucus production, eosinophilic inflammation, and Th2 responses upon reinfection [53,54]. Follow-up studies on the impact of reinfection may reveal genotype differences and development of RSV-specific features.

### 4.2. Differences in Viral Presence in Ts65Dn Mice May Be Linked to Genotype Related Immune Response

The immune fingerprint of Ts65Dn mice showed small signs of immune alterations and an altered systemic immune response with slightly lower CD8+ T cells and B cells, similar to the human situation [28,30]. These immune alterations observed in individuals with Down syndrome may, in part, explain the increased susceptibility for a LRTI.

Upon rhRSV-Luc infection, we observed a clear local immune response with an increase in total number of leukocytes in the lungs. The number of dendritic cells, natural killer T cells, and macrophages all increased in the lung over the duration of the rhRSV-Luc infection with peaks at seven days infection in both genotypes. A systemic neutrophilic inflammation is the predominant phenotype observed in infants with both mild and severe disease [55,56]. However, in the Ts65Dn mice, we did not observe a systemic neutrophilic response. Based on our results, we hypothesize that in this Ts65Dn mouse model, the low numbers of systemic monocytes may drive a less efficient adaptive immune response in Ts65Dn mice. However, we are aware that multiple immune cell types play a role in the innate immune response and that this cannot be attributed to a decrease in one single cell type. Interestingly, apart from the decrease in LY6C- monocytes, Ts65Dn mice presented with a significantly higher CD4+/CD8+ T cell ratio at baseline level, which further increased as infection prolonged. In-depth analysis indicated that systemic differences in CD4+/CD8+ T cell ratio were mainly determined by lower number of CD8+ Tn and CD8+ Tcm cells in the Ts65Dn mice. Therefore, low monocyte numbers might be related to an impaired systemic CD8+ T cell response in Ts65Dn mice, potentially making the Ts65Dn genotype more susceptible to recurrent infections. In combination with lower B cell counts in four days infected Ts65Dn mice, this may result, in part, in a less efficient adaptive immune response. Similar to these results, we observed a lower variation within the Ts65Dn infected group whereas some wild type mice demonstrated a photon lux below baseline level, potentially indicating a slightly faster viral clearance in wild type mice.

These results indicate that the Ts65Dn mouse model, presenting minor immune-alterations, can be used in preclinical research RSV-induced respiratory infections in DS. This model allows more investigations toward the DS-specific immunological mechanisms and assists in preclinical research for effective anti-viral therapy or a human RSV (hRSV) vaccine. This model opens the door to obtaining essential information on treatment or vaccine responses in the DS-specific population. In the future, these advances will allow for testing of tailored treatments or vaccine strategy for children with DS to improve the mortality and morbidity related to respiratory infections.

### 4.3. The Rationale behind Opting for the Ts65Dn Mouse Model, hRSV Strain and Instillation Route

As background for the RSV infection model, we chose the Ts65Dn mouse model as it is one of the most commonly used preclinical models of DS since it replicates around 55% of human chromosome 21 orthologous genes and is bred on a C57BL/6 background. We are aware that differences in genetic background may result in clinical differences and lung hRSV titres [57]. The most commonly studied and permissive mouse strains are the BALB/c and immunodeficient nude mice, which demonstrate weight loss and moderate bronchiolitis upon hRSV 2–20 strain exposure [58]. Although the immune responses of C57BL/6 mice are most similar to the human situation, C57BL/6 mice appear to be the most resistant to RSV [59]. Therefore, the C57BL/6 background of the Ts65Dn and wild type mice, used in this study, may result in a less permissive model to study hRSV infections in a DS-specific context. In addition, different RSV clinical isolates induce variable disease severity in BALB/c mice [60], thereby identifying the line 19 hRSV strain as most pathogenic. Similar to the phenotype we observed, they highlight that the hRSV Long strain does not induce airway mucus expression [60,61,62]. However, the use of a rhRSV-Luc strain allowed longitudinal and spatiotemporal follow-up of infection development by visualization of infected host cells in the nasal and thorax area using BLI.

We used young adult—six- to seven-week-old mice which may not entirely resemble the target human (<2 year at initial infection) population since it mainly affects children <2 years of age. We chose intranasal, rather than intratracheal instillation of rhRSV-Luc, considering that the intranasal inoculation route mimics the clinical situation. As the current hypothesis presents that structural anomalies of the nasal compartments and upper airways may be, in part, responsible for retaining more viral particles, therefore inducing the risk for severe LRTI in individuals with DS [63], we did not want to bypass the upper airways. By doing so, our model is maximally relevant to study hRSV infection in the context of DS.

## 5. Conclusions

This study presents a novel model of hRSV infection in a DS-specific mouse model. Using longitudinal non-invasive imaging and immunological analysis we observed that a profound and productive rhRSV-Luc respiratory infection results in a mild phenotype in wild type and Ts65Dn mice. Our data are indicative for immunological alterations in the Ts65Dn mice compared to WT mice when exposed to RSV. This study highlights the potential of this imaging-supported Ts65Dn mouse model of LRTI to be used in preclinical research for RSV-induced respiratory infections in the context of DS. Hence, our study paves the way to implement this RSV-induced LRTI Ts65Dn mouse model in research studies and may be used as a tool for therapeutic and vaccine testing to capture immune-specific responses that can advance tailored treatment strategies and improve mortality and morbidity in the DS population.

## Data Availability

All relevant data can be found within the article and its supplementary information. Any additional information required to reanalyze the data reported in this paper is available from the lead contact upon request.

## Supplementary Materials

The following supporting information can be downloaded at: https://www.mdpi.com/article/10.3390/v15040993/s1, Table S1. Antibodies used for flow cytometry; Figure S1: The gating strategy for the myeloid panel on flow cytometry in the lung and spleen; Figure S2: The gating strategy for the lymphoid panel on flow cytometry in the lung and spleen; Figure S3: BAL fluid total cell count and alveolar macrophages; Figure S4: Flow cytometric analysis of the lung immunological status in wild type and Ts65Dn mice; Figure S5: Proportions of CD4+ and CD8+ T cell populations in the lung of wild type and Ts65Dn mice throughout rhRSV-Luc infection; Figure S6: Proportions of CD4+ and CD8+ T cell populations in the spleen of wild type and Ts65Dn mice throughout rhRSV-Luc infection.
