# Supplementary material for: A Multimodal Imaging-Supported Down Syndrome Mouse Model of RSV Infection
Source: Viruses. 2023 Apr 18;15(4):993. doi: 10.3390/v15040993 (PMC10144178; doi:10.3390/v15040993)
Supplement: Supplementary file 1 [file viruses-15-00993-s001.zip › viruses-2345235-supplementary.pdf]

Supplementary Materials:

The following supporting information can be downloaded at: www.mdpi.com/xxx/s1,

Table S1. Antibodies used for flow cytometry;
Figure S1. The gating strategy for the myeloid panel on flow cytometry in the lung and spleen;
Figure S2. The gating strategy for the lymphoid panel on flow cytometry in the lung and spleen;
Figure S3. BAL fluid total cell count and alveolar macrophages;
Figure S4. Flow cytometric analysis of the lung immunological status in wild type and Ts65Dn mice;
Figure S5. Proportions of CD4+ and CD8+ T cell populations in the lung of wild type and Ts65Dn mice throughout rhRSV-Luc infection;
Figure S6. Proportions of CD4+ and CD8+ T cell populations in the spleen of wild type and Ts65Dn mice throughout rhRSV-Luc infection.

Supplementary Table S1: antibodies used for flow cytometry

Antigen	Fluorophore	Company
Lymphoid panel (100 000 cells read)		
CD3	FITC	eBioscience
CD8	PerCP-Cy5.5	eBioscience
CD44	PE	Biolegend
NK1.1	PE-Cy7	eBioscience
CD4	APC-eFluor 780	eBioscience
CD62L	APC	eBioscience
B220	BV786	BD
CD45	BUV395	BD
Live/dead	Zombie Aqua™ Fixable Viability Kit	Biolegend
Myeloid panel (200 000 cells read)		
CD45	FITC	Biolegend
CD103	PerCP-eFluor 710	eBioscience
CD11c	PE-Cy7	Biolegend
CD64	PE	Biolegend
F4/80	PE	eBioscience
CD24	PE-CF594	Biolegend
SiglecF	eFluor 660	eBioscience
Ly6G	Alexa fluor 700	BD
Ly6C	APC-Cy7	BD
CD11b	eFluor 450 or PerCP-Cy5.5	eBioscience
MHC-II	Horizon v500	BD
CD3	BV650	Biolegend
CD19	BV650	Biolegend
NK1.1	BV650	Biolegend
Live/dead	Zombie UV™ Fixable Viability Kit	Biolegend

Supplementary Figure S1 - gating strategy for myeloid panel

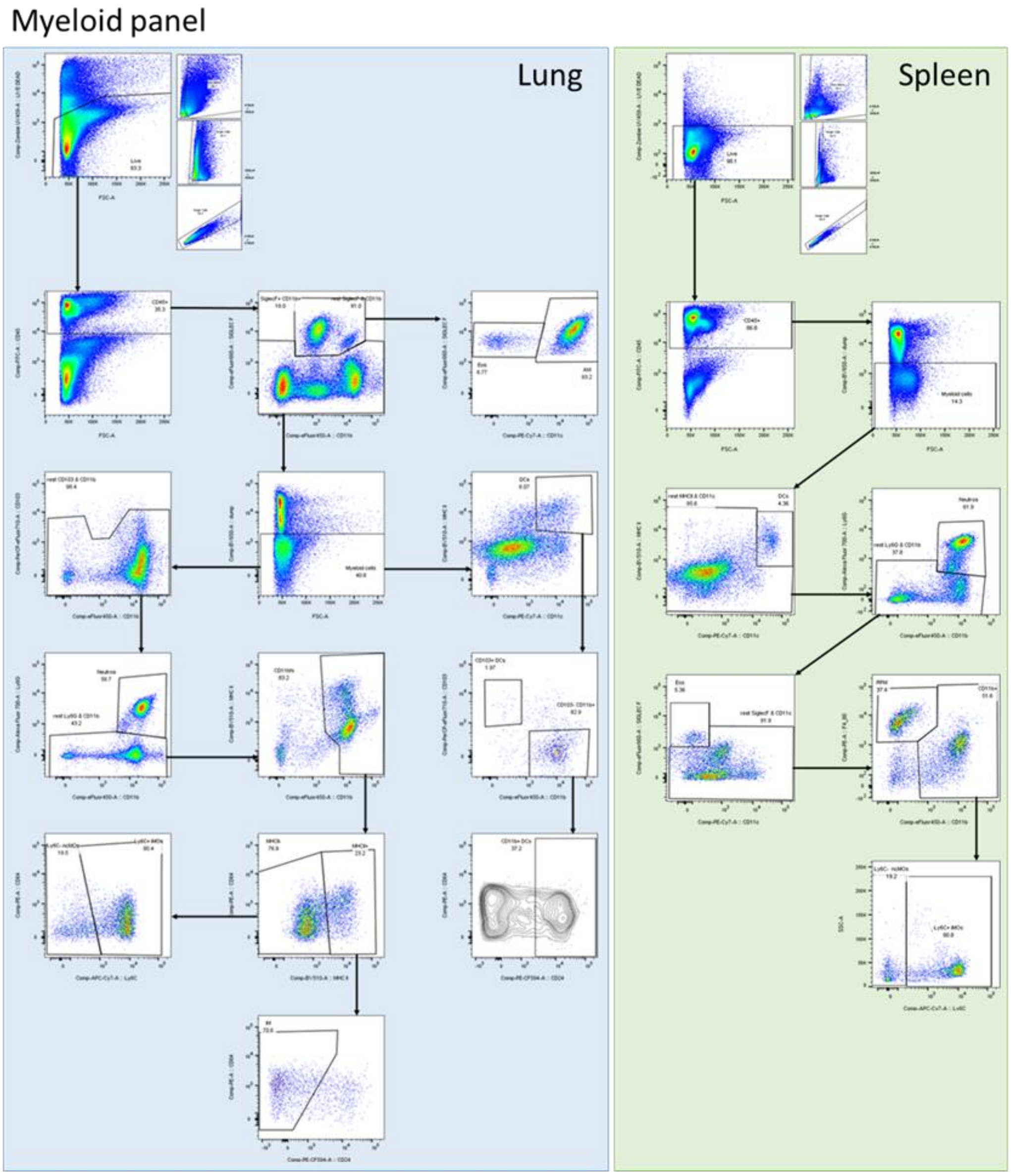

Supplementary Figure S1: The gating strategy for the myeloid panel on flow cytometry in the lung and spleen seven days post-infection. Lung and spleen cells were isolated, stained for myeloid marker and analyzed using flow cytometry. After the exclusion of red blood cells, debris and doublets of cells, all live cells (LD) were gated. The leukocytes were identified by CD45 staining, following live/death staining.

Supplementary Figure S2 – gating strategy for lymphoid panel

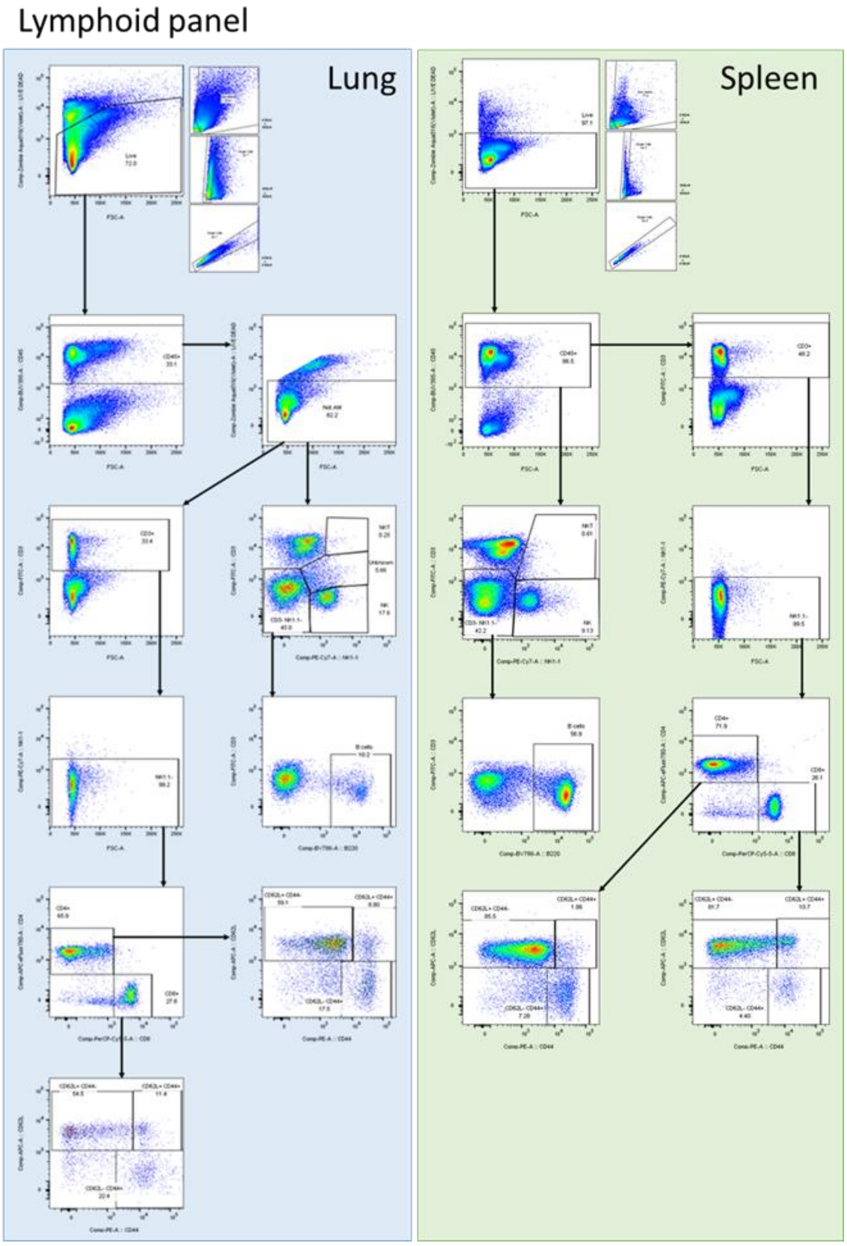

Supplementary Figure S2: The gating strategy for the lymphoid panel on flow cytometry in the lung and spleen seven days post-infection. Lung and spleen cells were isolated, stained for lymphoid markers and analyzed using flow cytometry. After the exclusion of red blood cells, debris and doublets of cells, all live cells (LD) were gated. The leukocytes were identified by CD45 staining following live/death staining.

Supplementary Figure S3 – BAL total cell count and alveolar macrophages

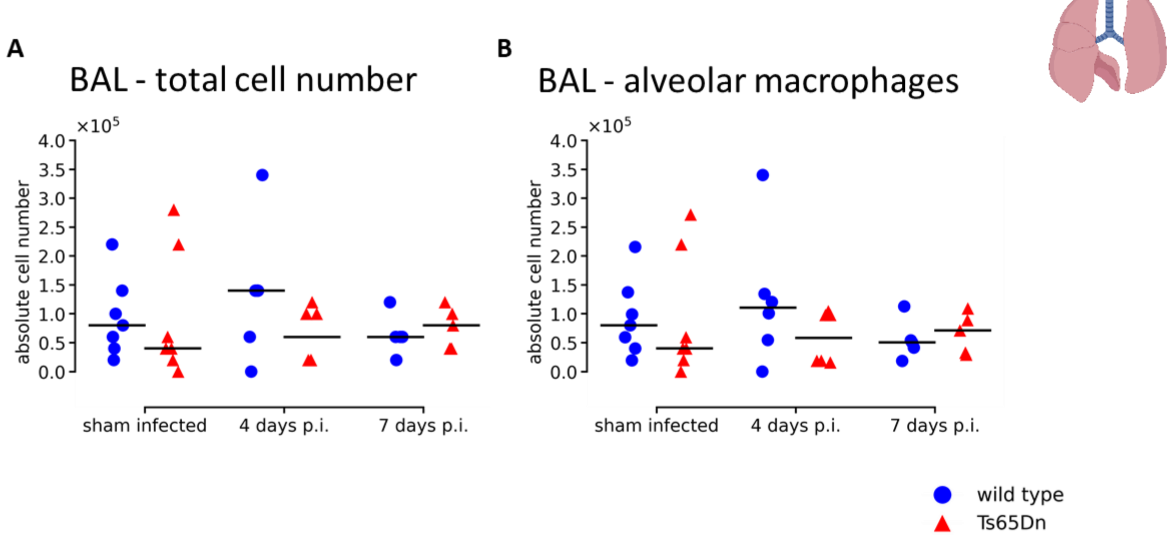

Supplementary Figure S3: BAL fluid total cell count and alveolar macrophages. (A) Analysis of the total cell count in BAL fluid and (B) number of alveolar macrophages in wild type (blue circle) and Ts65Dn (red triangle) mice at each time point and expressed as individual values and group median. *p <0.05, **p <0.01, ***p <0.001, ****p <0.0001.

Supplementary Figure S4 – Lung myeloid cells

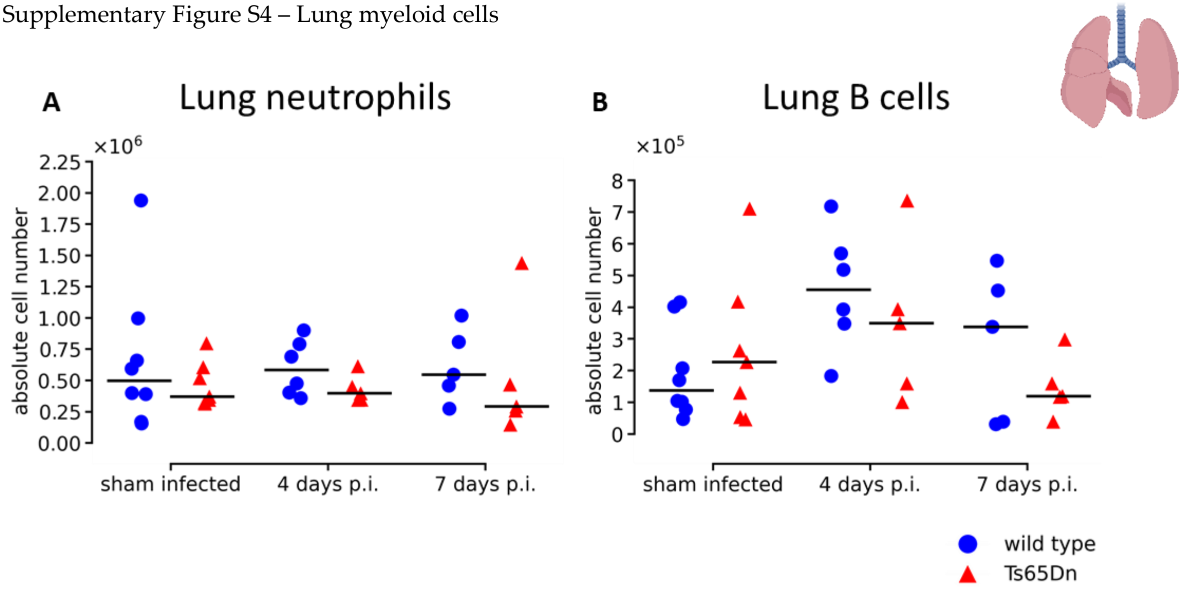

Supplementary Figure S4: Flow cytometric analysis of the lung immunological status in wild type and Ts65Dn mice after sham, four days or seven-days rhRSV-Luc infection. (A) the absolute number of lung neutrophils and (B) lung B-cells were presented in wild type (blue circle) and Ts65Dn (red triangle) mice at each time point and expressed as individual values and group median. *p <0.05, **p <0.01, ***p <0.001, ****p <0.0001.

Supplementary Figure S5 – Lung T cells

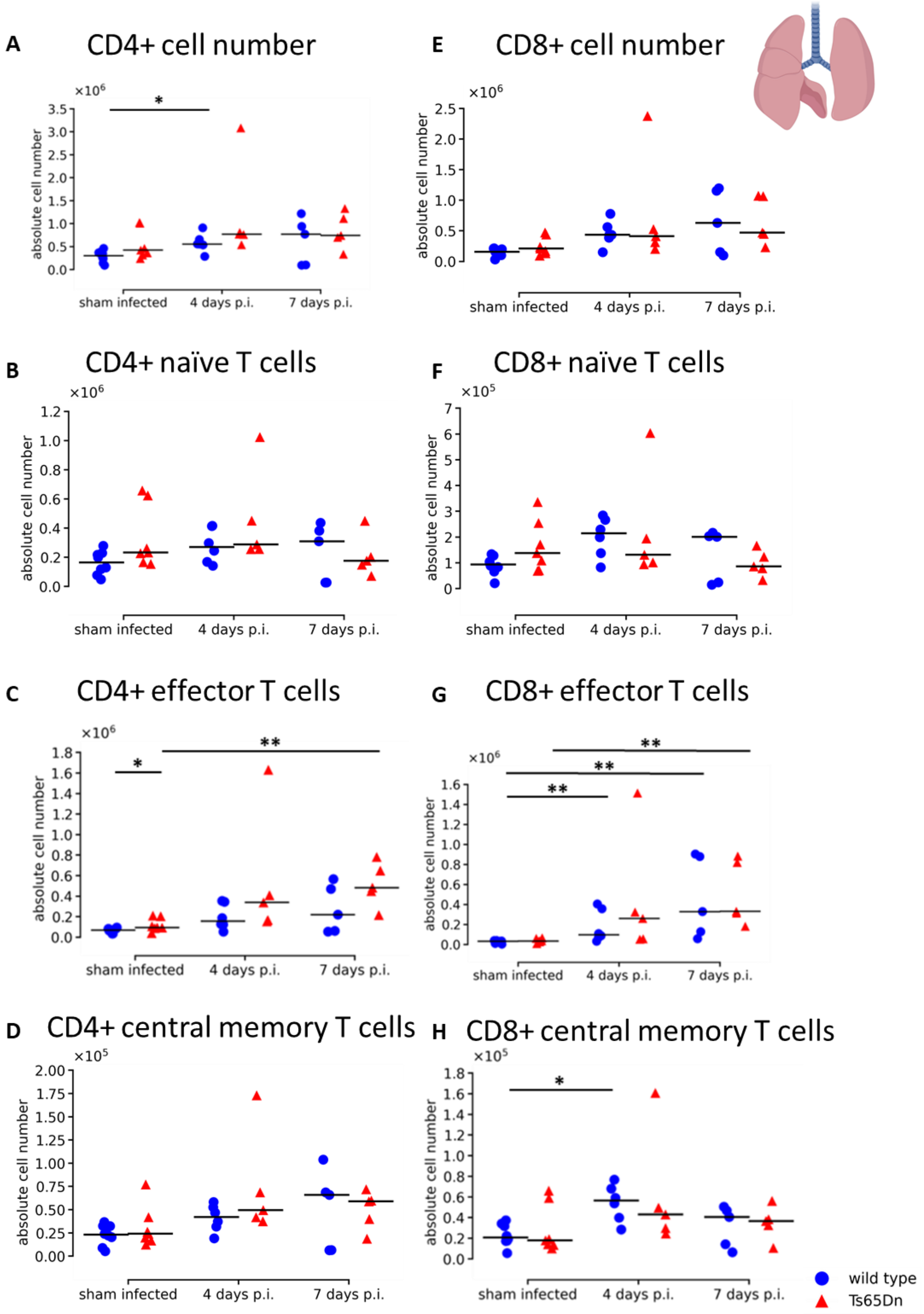

Supplementary Figure S5: Proportions of CD4+ and CD8+ T cell populations in the lung of wild type and Ts65Dn mice throughout rhRSV-Luc infection. (A). The absolute number of CD4+ T cells (CD45+, CD3+, CD4+), (B) CD4+ naïve T cells (CD44− CD62L+), (C) CD4+ effector T cells (CD44+ CD62L−) and (D) CD4+ central memory T cells (CD44+ CD62L+) and the absolute number of (E) CD8+ T cells (CD45+, CD3+, CD8+), (F) CD4+ naïve T cells (CD44− CD62L+), (G) CD4+ effector T cells (CD44+ CD62L−) and (H) CD8+ central memory T cells (CD44+ CD62L+) of wild type and Ts65Dn mice were presented as individual values with group median for sham infected, four and seven-day post infection in lung digestion. *p <0.05, **p <0.01, ***p <0.001, ****p <0.0001.

Supplementary Figure S6 – Spleen T cells

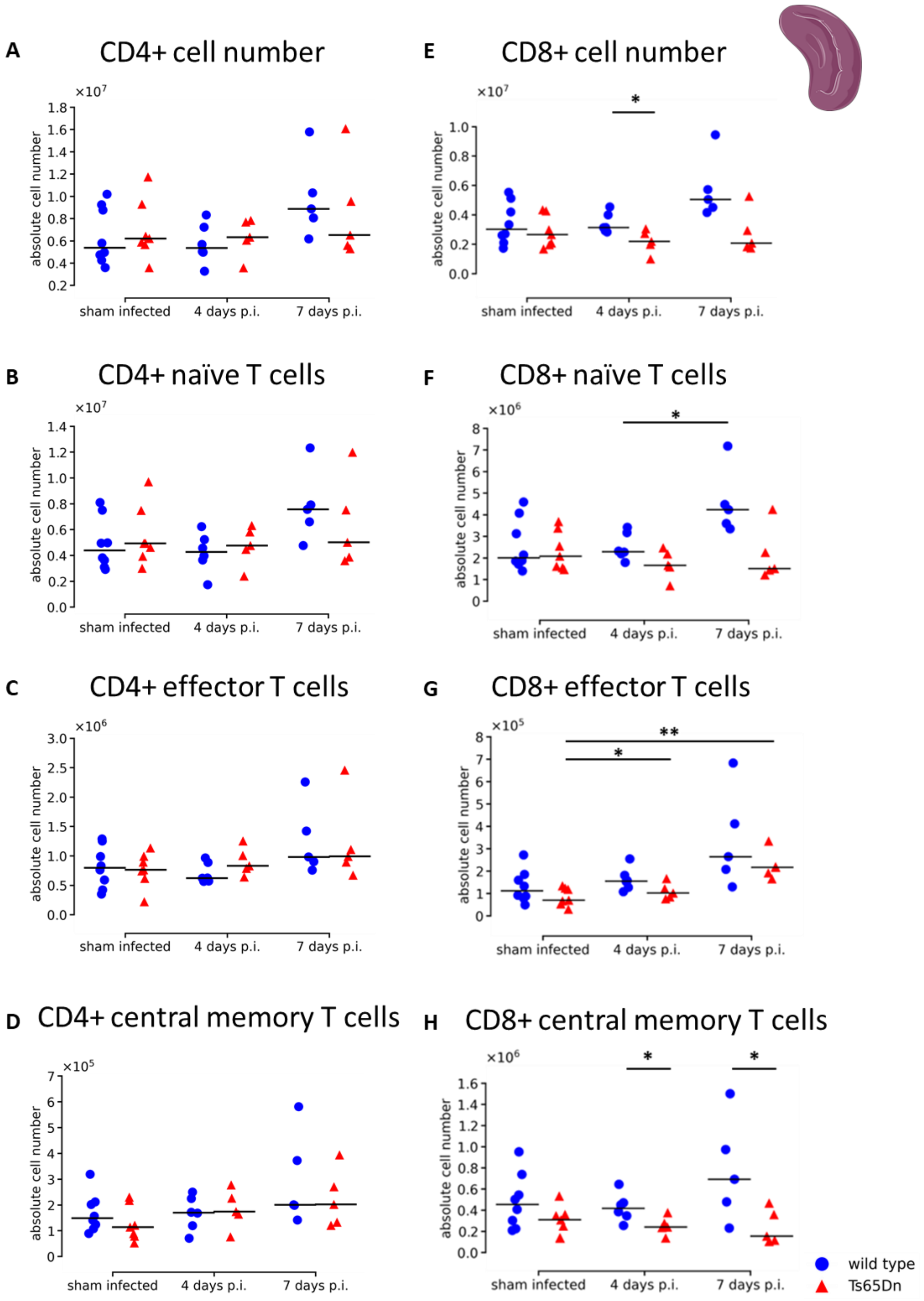

Supplementary Figure S6: Proportions of CD4+ and CD8+ T cell populations in the spleen of wild type and Ts65Dn mice throughout rhRSV-Luc infection. (A). The absolute number of CD4+ T cells (CD45+, CD3+, CD4+), (B) CD4+ naïve T cells (CD44− CD62L+), (C) CD4+ effector T cells (CD44+ CD62L−) and (D) CD4+ central memory T cells (CD44+ CD62L+) and the absolute number of (E) CD8+ T cells (CD45+, CD3+, CD8+), (F) CD4+ naïve T cells (CD44− CD62L+), (G) CD4+ effector T cells (CD44+ CD62L−) and (H) CD8+ central memory T cells (CD44+ CD62L +) of wild type and Ts65Dn mice were presented as individual values with group median for sham infected, four and seven-day post-infection in spleen digestion. *$p < 0.05$, **$p < 0.01$, ***$p < 0.001$, ****$p < 0.0001$.
